# Emotional Memory in Post-traumatic Stress Disorder: A Systematic PRISMA Review of Controlled Studies

**DOI:** 10.3389/fpsyg.2019.00303

**Published:** 2019-03-05

**Authors:** Florence Durand, Clémence Isaac, Dominique Januel

**Affiliations:** ^1^Unité de Recherche Clinique (URC), EPS Ville Evrard, Neuilly-sur-Marne, France; ^2^Laboratory of Neuropsychology and Psychopathology, University of Paris, Saint-Denis, France

**Keywords:** Post-traumatic stress disorder, PTSD, emotion, memory, emotional memory, attentional bias, interaction cognition-emotion, interaction memory-emotion

## Abstract

**Background:** Emotional memory is an adaptive process that improves the memorization of emotional events or stimuli. In Post-Traumatic Stress Disorder (PTSD), emotional memory may be altered, which in turn may affect symptoms. Having a clearer view of the processes of interaction between memory and emotional stimuli in PTSD may improve our knowledge of this disorder, and could create new therapeutic management tools. Thus, we performed a systematic review of the evidence of specific emotional memory in PTSD patients.

**Method:** Following PRISMA guidelines, a systematic review of MEDLINE, PsycInfo, and ScienceDirect was undertaken to identify controlled studies on emotional memory that used cognitive tasks on PTSD patients. The initial research was conducted from June 2017 to July 2017, and search terms included: Post-Traumatic Stress Disorder; PTSD; emotional memory; emotion; emotional; memory; and episodic memory.

**Results:** Eighteen studies reporting on 387 PTSD patients met the eligibility criteria. Among the studies selected, 11 observed specific memory processing in PTSD patients, such as a greater memorization of negative information, or a trend to false recognition of negative information. In addition, attentional and inhibition processing seem to play an important role in emotional memory in PTSD sufferers. Furthermore, other studies that did not find behavioral differences between PTSD and control groups nevertheless showed differences in both specific cerebral activities and neurohormone levels during emotional memory tasks.

**Conclusion:** This review has several limitations, including a limited number of controlled studies, small sample sizes, different tasks and methods. Nevertheless, the results of this systematic review provide interesting information on emotional memory for clinicians and researchers, as they seem to highlight facilitated memory processing for negative information in PTSD patients. This topic needs further controlled studies with sensitive behavioral tasks. Also, future studies may evaluate emotional memory after symptom amelioration.

## Introduction

Emotional memory refers to the attribution of emotional significance to a stimulus or event, promoting memory retention. Emotional events or stimuli are usually better memorized than neutral ones (Christianson, 2014). This interaction between emotion and memory helps individuals adapt to their environment, for example in order to avoid dangerous situations in the future. However, after exposure to terrifying events, the emotional memory can be altered as observed in Post-Traumatic Stress Disorder [PTSD].

PTSD is a mental illness marked by a specific clinical syndrome including symptoms, which last more than 1 month, of intrusions, avoidance, alterations of mood and cognition, and hyperarousal (American Psychiatric Association, 2013). PTSD occurs following a traumatic event that elicits fear, horror, and/or helplessness which may include actual or threatened death, bodily injury, or physical harm to one's self or other people. The lifetime prevalence of PTSD is between 4 and 6% among trauma-exposed patients worldwide (Koenen et al., 2017). PTSD is a particularly interesting disorder because of the development of a stronger and a more emotional memory of the traumatic event. One PTSD symptom involving memory and emotional processing is intrusive memories that consist of involuntary images accompanied by a high level of physiological arousal and are experienced as a reliving of the original traumatic event (Brewin et al., 1996). Patients relive the trauma, with all its emotional intensity, as though it were occurring in the present: memories are intrusive, extremely distressing, and occur on a regular basis. These memories are often reported by PTSD patients, and are triggered by the presence of elements that recall the traumatic event and consequently impact their daily life. Thus, emotions linked with psychological trauma alter or restrict cognitive processing, and can have long-term cognitive effects (Vasterling et al., 1998; Hayes et al., 2012). Indeed, PTSD patients tend to complain about cognitive functions, notably memory difficulties, which impact their daily functioning (Tapia et al., 2007; Moore, 2009). Understanding how emotion interacts with memory, whether the emotional stimuli is related to a trauma or not, could therefore be very beneficial to understand this disorder and suggest improvement in treatment for PTSD patients. From a neuroanatomical point of view, several researchers have emphasized the structural and functional modifications of brain structures in PTSD patients, such as the amygdala and hippocampus, which are also involved in memory and emotional processing (Kitayama et al., 2005; Shin and Liberzon, 2010; Morey et al., 2012).

Over the last few years, behavioral studies have been conducted to understand the influence of emotion on the memory function of PTSD patients, but until this paper, no review has synthesized the results. Furthermore, comparing emotional memory processes in PTSD patients with control participants could provide insight into the specificities of “normal” emotional memory processing. This systematic review therefore aims to review controlled studies that explore the influence of emotion on memory function through memory tasks, comparing emotional to neutral stimuli in PTSD patients.

## Method

This systematic review of the literature was performed according to PRISMA systematic review guidelines (Moher et al., 2009). Databases included MEDLINE, PsycInfo, and ScienceDirect.

### Eligibility Criteria

We included all studies that evaluated emotional memory via cognitive tasks in patients suffering from a Post-Traumatic Stress Disorder (PTSD) without age restriction.

Studies were eligible if they included individuals with a diagnosis of PTSD, according to the version of the Diagnostic and Statistical Manual of Mental Disorder in use at the time of each study, and if they included a control group. We selected studies that assessed emotional memory using cognitive tasks which compared the memorization of emotional compared to neutral stimuli (excluding autobiographical memory tasks). The studies also had to be written in English without any restriction on publication year. In addition, we restricted our selection to peer-reviewed articles (excluding poster presentations, oral communications, letters to the editor, and chapters of books).

Search terms were selected to target our population (Post Traumatic Stress Disorder) and our subject of interest by using several key-words: emotional memory, emotion, memory, emotional, episodic memory (see Appendix for search method for the MEDLINE database). The systematic review was not registered on PROSPERO.

### Selection Methods

One reviewer (FD) searched using the terms cited above (in the eligibility criteria) to identify relevant emotional memory studies that involved PTSD patients.

Titles and abstracts were screened by FD in order to remove duplicates. Then titles and abstracts were assessed independently by two reviewers (FD and CI) and articles were excluded if both reviewers decided that the articles clearly did not meet criteria. In case of disagreement, the two reviewers had to reach a consensus.

Then, the full text versions of remaining articles were assessed independently against inclusion and exclusion criteria by the same two reviewers. In case of disagreement, the two reviewers had to reach a consensus.

### Data Collection Process

Information was extracted and collected using a spreadsheet under the following headlines: first author, title, year of publication, number of patients and participants, age, gender, study design, assessments, results, and risk of bias. Risk of bias was assessed by adapting the Cochrane Risk of Bias tool, both at outcome and at study level (Higgins et al., 2011). If remuneration for participants had not been specified we assumed that no remuneration was handed out. If blind evaluation was not specified we assumed that assessors was not blind to the diagnosis of participants. If information about the support of the memory task was not specified we concluded that the memory task was not a computerized task.

## Results

The initial search on PudMed, PsycInfo, and ScienceDirect was conducted from June 2017 until July 2017 and provided 733 potentially eligible studies. Once duplicates had been removed, a total of 389 titles and abstracts were screened.

We excluded 317 articles after assessing their title and abstract because they clearly didn't meet our eligibility criteria: 85 studies had a different population (acute PTSD, healthy participants, other pathologies), 118 reports did not assess emotional memory with a cognitive task, 114 did not present study results.

**Figure 1:**
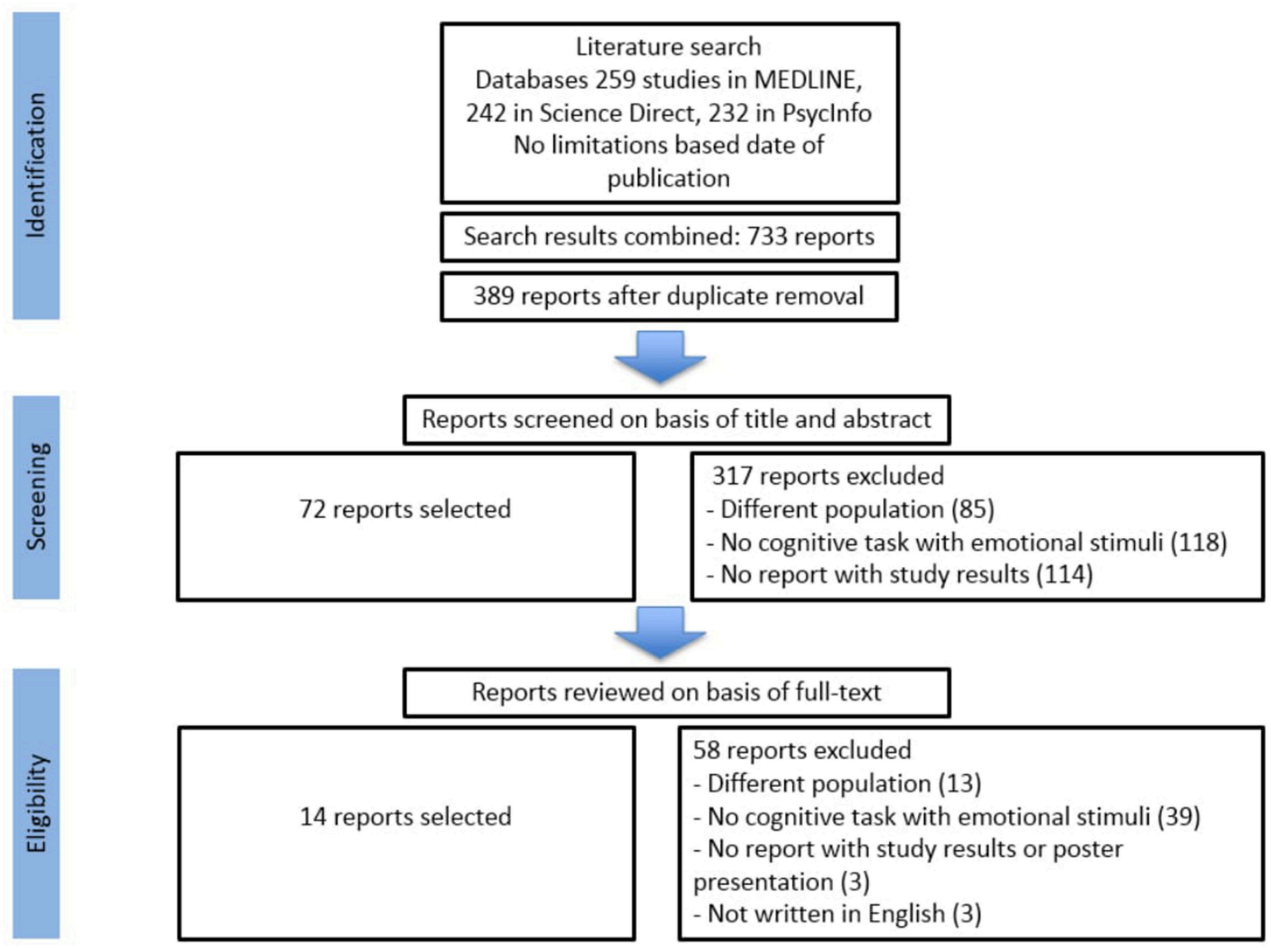
Summary of literature search, adapted from PRISMA (Moher et al., 2009).

After full text reviews of the 72 remaining reports, 58 were excluded: 13 studies had a different population, 39 reports did not assess emotional memory with a cognitive task, 3 studies were not written in English, and 3 did not present study results or were poster presentations. As a result of the selection criteria, 13 papers were selected. Furthermore, 5 additional papers were also identified through a manual search (either online or by searching through literature). Finally, 18 papers were selected for the systematic review (see Figure 1).

### Characteristics of the Studies

Table 1 shows characteristics of included studies and main behavioral results. Seven studies used cognitive tasks with picture stimuli, 10 studies used word stimuli, and one with picture and word stimuli.

**Table 1:** Studies' characteristics and behavioral results of emotional memory in PTSD.

**References**	**Groups**	**Trauma type**	**Sex/Age**	**Stimuli**	**Tasks**	**Behavioral results**
Baumann et al., 2013	12 PTSD 13 HC	Migrants and refugees	M&F 32.3 (9.28)	Non trauma-related emotional information pictures	Item-method directed forgetting task following by a recognition task	Emotional/neutral: PTSD = HC
Bremner et al., 2003	10 PTSD 11 HC	Childhood sexual abuse	F 40 (6)	Negative word pairs that have fear-related or life-threatening content and neutral word pairs	Word-pair task	Emotional/neutral: PTSD = HC
Brohawn et al., 2010	18 PTSD 18 TE	Varied	M&F 28.2 (7.8)	Negative, neutral, and positive pictures	Visual memory recognition task	Emotional/neutral: PTSD = TE
Chemtob et al., 1999	16 PTSD 27 TE 20 HC 16 Psy	Vietnam combat veteran	M 45.81 (6.89)	Combat related and neutral words and pictures	free recall for words and recognition task for pictures	*Free recall*: combat related words/neutral words: PTSD > others groups; PTSD were more likely to produce combat related word as first word they recalled *Recognition:* combat related/neutral pictures: PTSD = others groups
Golier et al., 2003	31 PTSD 17 TE 34 HC	Holocaust survivors	F 67.7 (5.6)	Low associate pairs consisted of a Holocaust-related words paired with a neutral word (unrelated words), and high associate pairs consisted of neutral word pairs (moderatly related words)	Word-pair task and word-stem completion test	*Word-pair task*: PTSD patients recalled more words from the Holocaust-related pairs than from neutral word pairs, compared to control groups W*ords stem completion:* holocaust-related word/neutral: PTSD = TE = HC
Guillery-Girard et al., 2013	25 PTSD 24 HC	NS	M&F 13.6 (1.13)	Neutral and negative non-trauma related pictures (perceptual and conceptual pictures)	Oddball task following by a recognition task	Negative/neutral: PTSD+ = control; PTSD– ≠ control: PTSD-: negative perceptuals pictures < neutral & negative conceptual pictures; more false negative conceptual recognitions
Herzog et al., 2017	28 PTSD 28 TE 28 HC	Childhood sexual, physical abuse	F 30.61 (9.99)	Negative trauma-related words, negative words, neutral words and color words	Emotional Stroop task following by free recall and recognition task	Emotional/neutral: PTSD = HC = TE
McNally et al., 1998	14 PTSD 12 TE 12 HC	Childhood sexual abuse	F 41.6 (8.0)	Trauma related, positive, and neutral words	Directed forgetting task then free recall	PTSD/TE and HC: Direct forgetting effect only for positive and neutral words.
Mickley Steinmetz et al., 2012	25 PTSD 27 TE 25 HC	Varied	M&F 39.68 (14.28)	Images of positive, negative and neutral items onto neutral background scenes	Memory recognition task for the items and backgrounds separately	Memory trade off: PTSD > control groups
Moradi et al., 2000	24 PTSD 25 HC	Road traffic or personal violence events	M&F 13.0 (2.8)	Positive, negative, neutral words	Free recall and recognition task	*Free recall:* negative words: PTSD = HC; positive words: PTSD < HC; Neutral words: PTSD < HC; *Recognition task*: PTSD = HC
Nicholson et al., 2014	18 PTSD 20 TE 20 HC	Varied	M&F 30.72 (13.42)	Negative, neutral and positive pictures stimuli	Delayed memory free recall task after two days	Negative /positive and neutral**:** TE and PTSD > HC; TE> control; PTSD = HC
Patel et al., 2016	11 PTSD 11 TE	Varied	M&F 31.45 (10.72)	Negative arousing, negative non-arousing, positive arousing, positive non-arousing and neutral pictures	Memory recognition task	Emotional/neutral: PTSD = TE
Tapia et al., 2012	15 PTSD 15 HC 15 A&D	Varied	M&F 24.6 (8.3)	Positive, negative, neutral words	Recognition task with RKG paradigm	PTSD = other groups, but PTSD/HC were more likely to “remember” negative words than to “know” it
Thomaes et al., 2013	30 PTSD 25 HC	Childhood sexual and/or physical abuse	F NR	Neutral and negative words	Declarative memory recognition task	Negative/neutral: PTSD = HC; PTSD: False alarm to negative words (trend)
Vrana et al., 1995	42 PTSD 15 TE	Vietnam War trauma	M 44.8 (3)	Vietnam War-related words with neutral and negative meaning, general negative words and neutral words	Emotional Stroop task following by a free recall then a recognition task	*Free recall:* Vietnam-related and negative words /neutral words: PTSD > control *Recognition:* Vietnam-related and negative words/ neutral words: PTSD = control
Whalley et al., 2009	16 PTSD 16 TE 16 D	Varied	M&F 36.8 (7.6)	Pictures of object superimposed on an emotional background context non trauma- related	Visual memory recognition task	Emotional/neutral: PTSD = control
Zeitlin and McNally, 1991	24 PTSD 24 TE	Vietnam combat veteran	M 41.13 (2.98)	Negative (combat, social threat), positif and neutral words	Word completion task (implicit memory), cued recall (explicit memory)	*Cued recall*: negative words: TE = PTSD; neutral and positive words: TE>PTSD. *Word completion*: PTSD/TE completed more combat-related words than social threat, positive and neutral words; PTSD/TE: no differences for other words type.
Zoellner et al., 2003	28 PTSD 28 HC	Sexual/nonsexual assault	F NR	Negative threat-related, positive, and neutral words	Item-cued directed forgetting task with mood induction following by free recall and recognition task	Emotional/neutral: PTSD = HC

*PTSD, Post-Traumatic Stress Disorders patients; PTSD–, PTSD with attentional deficit; PTSD+, PTSD with similar or superior attentional abilities to healthy participants; TE, Trauma-Exposed participants; HC, Healthy control participants; D, Depressed participants; Psy, patients with varied psychiatric diseases; A&D, patients with anxiety and depression disorders; NR, Not Reported; F, Female; M, Male; FA, False alarms; “Emotional/neutral”: when we compare recall of emotional stimuli to the recall of neutral stimuli*.

Among studies involving tasks with word stimuli: two studies used recognition words after an emotional Stroop task (Vrana et al., 1995; Herzog et al., 2017); two studies used word-pair tasks (Bremner et al., 2003; Golier et al., 2003); four studies used a verbal declarative memory task with free recall and/or recognition (Chemtob et al., 1999; Moradi et al., 2000; Tapia et al., 2012; Thomaes et al., 2013); two others used an item-cued directed forgetting task (McNally et al., 1998; Zoellner et al., 2003), one study used cued recall (Zeitlin and McNally, 1991) and two studies used word stem completion (Zeitlin and McNally, 1991; Golier et al., 2003).

Among studies involving tasks with picture stimuli: five studies used a recognition memory task (Chemtob et al., 1999; Whalley et al., 2009; Brohawn et al., 2010; Mickley Steinmetz et al., 2012; Patel et al., 2016); one study used a recognition memory task after an oddball task (Guillery-Girard et al., 2013); one study used an item-method directed forgetting task (Baumann et al., 2013); and another study used a delayed memory free recall test (Nicholson et al., 2014).

Selected reports included a total of 387 PTSD patients, 215 Trauma-Exposed participants without PTSD (TE), 16 depressed patients, 15 depressed/anxious patients, 16 psychiatric patients (other than PTSD or psychotic disorder), and 280 Healthy Control participants (HC). Six studies included only women, three studies included only men, and nine studies included both. Sixteen studies included only adult PTSD patients, and two studies included only adolescent and/or child PTSD patients (Moradi et al., 2000; Guillery-Girard et al., 2013). Eleven studies involved patients with one or two specific types of traumatic event (Zeitlin and McNally, 1991; Vrana et al., 1995; McNally et al., 1998; Chemtob et al., 1999; Moradi et al., 2000; Bremner et al., 2003; Golier et al., 2003; Zoellner et al., 2003; Baumann et al., 2013; Thomaes et al., 2013; Herzog et al., 2017), six studies involved patients with different traumatic events (Whalley et al., 2009; Brohawn et al., 2010; Mickley Steinmetz et al., 2012; Tapia et al., 2012; Nicholson et al., 2014; Patel et al., 2016), and one study did not specify (Guillery-Girard et al., 2013).

Among 18 reports, eight studies used two control groups. One study compared PTSD patients to patients with current major depression, as well as TE participants. Another study compared PTSD to HC and patients suffering from depression/anxiety. Six other studies compared PTSD patients with TE and HC.

Ten studies used one control group: four studies only compared PTSD patients with TE; and six studies only compared PTSD patients with HC.

Furthermore, six studies had additional neuroimaging results. Neural processes of emotional memory were assessed with functional Magnetic Resonance Imaging (fMRI), and one study used Positron Emission Tomographic (PET) imaging.

All 18 reports were published between 1991 and 2017.

### Study Results

Regarding studies with word stimuli, six studies used free recall. Four studies reported a greater memory for negative words in PTSD groups (Vrana et al., 1995; McNally et al., 1998; Chemtob et al., 1999; Moradi et al., 2000). Two studies observed that PTSD sufferers recalled a greater percentage of emotional words that were related to their specific trauma, compared to control groups (Vrana et al., 1995; Chemtob et al., 1999). Another study reported a direct forgetting effect for emotional and neutral words only in the PTSD group compared to the control groups (McNally et al., 1998). Finally, Moradi et al. (2000) observed that their PTSD group recalled an equal amount of negative words compared to HC, but fewer positive and neutral words. On the contrary, two studies did not report any differences: more emotional words (trauma-related or threat-related words) were remembered across all groups (Zoellner et al., 2003; Herzog et al., 2017).

Six studies used verbal recognition tasks. They did not report differences between PTSD and control participants (Vrana et al., 1995; Moradi et al., 2000; Zoellner et al., 2003; Thomaes et al., 2013; Herzog et al., 2017). Nevertheless, two of these studies reported interesting results. Thomaes et al. (2013) found a trend for PTSD groups to falsely recognize negative words compared to the control group (Thomaes et al., 2013). Furthermore, Tapia et al. (2012) used a recognition task with a Remember/Know paradigm, and found that PTSD patients were more likely to “remember” negative words than “know” them, compared to control groups.

Two studies used word-pair tasks to test emotional memory. One study observed that more deeply encoded neutral word pairs were recalled than deeply encoded negative emotional word pairs, more of which were in turn recalled than shallowly encoded neutral word pairs. In this study, PTSD and control groups produced the same results (Bremner et al., 2003). Another study used a word-pair task and observed that the PTSD group recalled significantly more words from trauma-related pairs than from neutral pairs, while there was no difference between both control groups. This signifies that PTSD participants are more sensitive to the effects of trauma-related content (Golier et al., 2003).

Two studies used word completion tasks. One reported that there was no difference between the PTSD and both control groups when recalling more Holocaust-related than neutral words (Golier et al., 2003). The other found that the PTSD group completed more combat-related words than TE, and these differences were not found for other types of words (Zeitlin and McNally, 1991). Zeitlin and McNally also used a cued recall task where they found that negative words were recalled equally in all groups, but neutral and positive words were less recalled in PTSD compared to control groups.

Regarding studies with picture stimuli, two studies used recognition tasks with complex images composed of either emotional or neutral items in the background and foreground (Whalley et al., 2009; Mickley Steinmetz et al., 2012). Whalley et al. used either positive, negative or neutral backgrounds, with only neutral items in the foreground. They observed that PTSD sufferers and control groups (depressed patients and HC), recognized old items equally accurately whatever the valence of the context in which they had been encoded. Item recognition was equally accurate, regardless of the emotional content of the background.

Depressed and PTSD groups remembered fewer words than the group of HC, regardless of emotional content in the images. On the contrary, Mickley Steinmetz et al. (2012) used neutral backgrounds with either emotional or neutral items in the foreground. This was to evaluate an “emotion-induced memory trade-off,” which is an increase in memory for emotional foreground items compared to neutral foreground items, combined with a decrease in memory for backgrounds associated with emotional items compared to neutral items. Mickley Steinmetz et al. (2012) reported a stronger emotion-induced memory trade-off for PTSD compared to control groups for both positive and negative foreground items. Also, a trend for false recognition of negative words was observed in the PTSD group compared to control groups.

Five other studies used different types of image-based recognition tasks (Chemtob et al., 1999; Brohawn et al., 2010; Baumann et al., 2013; Guillery-Girard et al., 2013; Patel et al., 2016). Four of them revealed no differences in the accuracy of emotional picture recognition compared to neutral picture recognition in PTSD groups and control groups. Only Guillery-Girard et al. (2013) reported that PTSD adolescents with an attentional deficit recognized fewer negative perceptual targets and produced more false negative conceptual recognitions than controls. Nevertheless, PTSD sufferers with attentional capacities similar or superior to control participants had similar performance to controls in the recognition task.

One study (Nicholson et al., 2014) used a free recall task, and observed that TE and PTSD groups recalled more negative images than the HC group, but there were no significant differences in the number of negative images recalled between PTSD and HC.

Furthermore, eight studies used stimuli that had been extracted from published databases (Chemtob et al., 1999; Whalley et al., 2009; Brohawn et al., 2010; Mickley Steinmetz et al., 2012; Tapia et al., 2012; Guillery-Girard et al., 2013; Nicholson et al., 2014; Patel et al., 2016), five studies used stimuli that had been extracted from previous studies (Zeitlin and McNally, 1991; Moradi et al., 2000; Zoellner et al., 2003; Thomaes et al., 2013; Herzog et al., 2017), four studies did not use any databases (Vrana et al., 1995; McNally et al., 1998; Bremner et al., 2003; Golier et al., 2003), and one study did not specify (Baumann et al., 2013).

Overall, six reports provided results on neuroimaging data. Three studies applied neuroimaging during encoding (Brohawn et al., 2010; Patel et al., 2016; Herzog et al., 2017), two studies during retrieval (Bremner et al., 2003; Whalley et al., 2009) and one study during encoding and retrieval (Thomaes et al., 2013).

During encoding of negative stimuli, Thomaes et al. (2013) observed greater activity in the Anterior Cingulate Cortex (ACC), the dorsomedial PreFrontal Cortex (dmPFC), and the hippocampus in PTSD patients relative to control participants during encoding of later-remembered negative words vs. baseline. Another study reported a positive correlation between activation in the left hippocampus and the left amygdala when PTSD patients recalled negative compared to neutral stimuli. This recall of negative stimuli was more prominent in PTSD sufferers compared to the TE group (Brohawn et al., 2010). Patel et al. (2016) were specifically interested in amygdala activity in PTSD patients compared to their control group. They observed that the BasoLateral Amygdala (BLA) in PTSD patients is preferentially activated during the successful encoding of negative pictures compared to positive pictures. Furthermore, a PTSD group × valence interaction revealed activation in the left precentral gyrus, the right amygdala and right hippocampus. A recent study observed a greater activation in the right dorsolateral PreFrontal Cortex (dlPFC), left ventromedial PFC (vmPFC), left dorsal ACC (dACC), and right insula among PTSD patients during a presentation of trauma-related words compared to control groups (Herzog et al., 2017). There was no significant difference between PTSD and HC participants when recalling negative stimuli (Thomaes et al., 2013). Another study observed a greater activation among PTSD patients in the frontal superior cortex and precuneus, as well as decreased activation in the left temporal superior cortex when recalling negative relative to neutral stimuli (Whalley et al., 2009). Another of the six reports observed a higher activation in the left ventrolateral PFC (vlPFC), extending to the lateral orbitofrontal cortex, in baseline PTSD patients compared to controls during false recognition of negative stimuli (Thomaes et al., 2013). Another study used PET scans during retrieval of emotional words: PTSD patients showed an increase in motor cortex activation, ACC, visual association cortex, inferior parietal lobule, frontal cortex, and temporal cortex, with decreased activation in the orbitofrontal cortex, ACC, and medial prefrontal cortex when recalling deeply encoded emotional words, compared to deeply encoded neutral words. There was also a decrease in the left fusiform, inferior temporal cortex, middle temporal cortex, and left hippocampus (Bremner et al., 2003).

Overall, only one study reported biological results (Nicholson et al., 2014). Despite the fact that their PTSD group did not show significant differences in the number of negative stimuli recalled, relative to the HC group, the authors observed that the PTSD group had a greater noradrenaline level in saliva samples relative to the HC group. No differences were observed in cortisol level between the two groups (Nicholson et al., 2014).

### Risk of Bias

Table 2 shows the main risks of bias within the selected reports.

**Table 2:** Risk of bias.

**References**	**Control group(s)**	**Criteria for group matching**	**Medication**	**Blinded: assessors/computerized task**	**Incomplete participant data**	**Selective outcome reporting**	**Conflict of interest**	**Remuneration**
Baumann et al., 2013	HC	Age, sex, country of origin	NR	No/Yes	NR	4 PTSD and 1 HC (low performance)	NR	Yes
Bremner et al., 2003	HC	Sex, age, YoE, IQ	Free of all medications	No/No	NR	NR	NR	No
Brohawn et al., 2010	TE	Age, YoE	Free of psychotropic medication	No/No	2 PTSD for imaging, 1 PTSD for memory test	3 TE (low performance)	NR	No
Chemtob et al., 1999	TE, HC, Psy	Trauma type, YoE, ethnicity	NR	No/Yes	only 76 participants completed the word-recall phase of the experiment.	NR	NR	Yes
Golier et al., 2003	TE, HC	Age, sex, the age at time of trauma	NR	No/No	NR	NR	NR	No
Guillery-Girard et al., 2013	HC	Age, attentional capacities	Free of psychotropic medication	No/Yes	4 HC (results were not recorded)	2 HC (low performance)	NR	No
Herzog et al., 2017	TE HC	Age, sex, YoE, handedness	In PTSD group	No/Yes	NR	NR	NR	Yes
McNally et al., 1998	TE, HC	Age, trauma type	NR	No/Yes	Missing data in demographic and psychometric data	NR	NR	NR
Mickley Steinmetz et al., 2012	TE HC	Age, sex, YoE, age at time of trauma, time since trauma	NR	No/No	3 participants (failure to complete the second part of the study)	NR	NR	No
Moradi et al., 2000	HC	Age, verbal IQ, reading ability	NR	No/Yes	NR	NR	NR	NR
Nicholson et al., 2014	TE, HC	Sex, trauma type, time since trauma	In PTSD and TE group.	No/No	NR	NR	NR	No
Patel et al., 2016	TE	Age, sex, YoE	In PTSD and TE group	No/No	NR	NR	NR	No
Tapia et al., 2012	HC, A&D	Age, education level	In PTSD and A&D groups	No/Yes	NR	NR	NR	NR
Thomaes et al., 2013	HC	Age, sex, handedness	In PTSD group only	No/Yes	2 PTSD and 4 HC (technical problem), and 1 PTSD (panicked during scanning)	2 PTSD and 5 HC (too many omissions)	NR	No
Vrana et al., 1995	TE	Sex, number of months in combat	In PTSD and TE groups	No/No	Free recall data were not collected for 5 PTSD patients	NR	NR	No
Whalley et al., 2009	TE, D	Age, sex, handedness, YoE	NR	No/No	NR	NR	NR	No
Zeitlin and McNally, 1991	TE	Trauma type	NR	No/NR	NR	NR	NR	Yes
Zoellner et al., 2003	HC	Sex	NR	No/Yes	NR	NR	NR	Yes

*PTSD, Post-Traumatic Stress Disorder patients; TE, Trauma-Exposed participants; HC, Healthy Control participants; D, Depressed participants; NR, Not Reported; YoE, Years of Education; IQ, Intelligence Quotient*.

Nine studies reported incomplete data or selective outcome reporting. Four studies removed some specific subjects' data from their analyses, due to performance below chance level, performance below one standard deviation, or many omissions (Brohawn et al., 2010; Baumann et al., 2013; Guillery-Girard et al., 2013; Thomaes et al., 2013). Thomaes et al. (2013) found that results were similar both before and after the selective removal of this data. However, removing the data could have modified the final emotional memory results for the three other studies. Furthermore, two studies had incomplete data because of technical issues, head movement, or panic during the scan (Brohawn et al., 2010; Thomaes et al., 2013); in four other studies, the performance of a few participants were not recorded (Vrana et al., 1995; McNally et al., 1998; Chemtob et al., 1999; Guillery-Girard et al., 2013); and two other studies excluded participants because of a failure to complete the memory task (Brohawn et al., 2010; Mickley Steinmetz et al., 2012). Finally, Thomaes et al. (2013) only analyzed words rated by participants as “certainly seen.” Both selective outcome reporting and incomplete data may have modified the quality of group matching.

Of the 18 reports included, only two studies matched control groups to PTSD groups for cognitive ability (Bremner et al., 2003; Guillery-Girard et al., 2013). PTSD patients could have had different cognitive abilities than HC or TE that may have biased emotional memory results. This is important to note because memory involves multiple cognitive functions (Cabeza et al., 2003; Bäuml et al., 2010).

Furthermore, nine publications did not report any information about patients' medication (Zeitlin and McNally, 1991; McNally et al., 1998; Chemtob et al., 1999; Moradi et al., 2000; Golier et al., 2003; Zoellner et al., 2003; Whalley et al., 2009; Mickley Steinmetz et al., 2012; Baumann et al., 2013), and six studies reported that some PTSD participants were taking psychotropic medication (Vrana et al., 1995; Tapia et al., 2012; Thomaes et al., 2013; Nicholson et al., 2014; Patel et al., 2016; Herzog et al., 2017). Medication may influence cognitive performance and emotional processing (Schmitt et al., 2001; Outhred et al., 2014), and therefore can be a risk of bias. Among these five studies, two studies tried to control or evaluate the effect of medication on behavioral results (Vrana et al., 1995; Thomaes et al., 2013).

In five of the 18 publications, authors reported that participants were paid for their participation. As an external reward can lower intrinsic motivation (Kruglanski et al., 1975), remuneration could have modified participants' performance. Among the 14 studies selected, none of the assessors were blind to the diagnosis of PTSD, but nine studies used computerized memory tasks (McNally et al., 1998; Moradi et al., 2000; Whalley et al., 2009; Tapia et al., 2012; Baumann et al., 2013; Guillery-Girard et al., 2013; Thomaes et al., 2013; Herzog et al., 2017). The use of computerized-memory tasks or blind assessors avoids confirmation bias. Finally, no conflict of interest was specified in the 18 reports.

## Discussion

### Cognitive Examination of Emotional Memory

Among 18 reports, 11 studies reported differences in emotional memory between PTSD patients and control groups.

Seven studies used free recall tests to assess emotional memory (Vrana et al., 1995; McNally et al., 1998; Chemtob et al., 1999; Moradi et al., 2000; Zoellner et al., 2003; Nicholson et al., 2014; Herzog et al., 2017). Two of them did not find any difference in emotional memory between PTSD groups and control groups (Zoellner et al., 2003; Herzog et al., 2017), but five others found a greater memory for negative stimuli in PTSD sufferers. Nicholson et al. (2014) used a delayed memory recall task. Two days after exposure to positive and negative emotional pictures, as well as neutral pictures, TE and PTSD patients recalled more negative pictures than HC. However, no significant differences were observed between the PTSD and TE groups. Contrary to this, Vrana et al. (1995), showed that emotional memory seems to be PTSD-related, and not trauma-related. During free recall, Vrana et al. observed greater recall of all negative words for their PTSD group compared to TE. The authors of the study used emotional words that were related to the Vietnam War, general words related to the Vietnam War, general emotional words, and control words. Chemtob et al. (1999) found similar results: PTSD sufferers recalled more combat-related words than neutral words compared to other groups (TE, HC, Psy groups). Furthermore, McNally et al. (1998) also found a greater memory among PTSD patients for negative words. They used a directed-forgetting task during encoding. Usually, participants exhibited a directed forgetting effect by recalling more “remember words” than “forget words.” In PTSD patients compared to TE and HC, this directed-forgetting effect was only observed for positive or neutral words, but not for negative ones. In contrast to this finding, Zoellner et al. (2003) also used a directed forgetting paradigm, but in this study participants had a mood induction procedure before word encoding. Immediate recall results showed that more threat-related words were recalled than neutral or positive words, without any difference between PTSD and HC. The difference between McNally et al. (1998) and Zoellner et al. (2003) could be linked to the use of mood induction in the latter. Finally, Moradi et al. (2000) reported a verbal memory deficit for PTSD patients, although for negative words in a free recall task, there was no difference between PTSD sufferers and HC. This verbal memory deficit shows that emotional memory could be specifically PTSD-related. Herzog et al. (2017) used an emotional Stroop task with trauma-related words, general negative words and neutral words. The authors then tested emotional memory with an immediate free recall test. Contrary to Vrana et al. (1995), who used the same encoding phase, Herzog et al. (2017) did not provide evidence for a greater memory of emotional stimuli among PTSD patients. These studies used different free recall tasks to assess emotional memory, and had different control groups or different characteristics in PTSD patient groups (e.g., sample size, gender, and traumatic event). For instance, Vrana et al. (1995) and Herzog et al. (2017) used the same procedure, although the former involved male participants and the latter included female participants. However, men and women have different emotional processing that can influence memory (Cahill et al., 2001).

To summarize, among these seven free recall studies, four suggest that PTSD is associated with specific emotional memory, while another observed that PTSD and TE are associated with specific emotional memory (Nicholson et al., 2014), and the two other studies did not find specific emotional memory performance in any group (Zoellner et al., 2003; Herzog et al., 2017). Overall, during a recall task, PTSD patients seem to memorize more negative information, whether this negative information is directly related to the trauma (McNally et al., 1998; Chemtob et al., 1999) or is generally negative (Vrana et al., 1995; Brohawn et al., 2010; Nicholson et al., 2014). This negative memory facilitation could be due to attentional bias, which generates focused attention on negative and trauma-related words for PTSD sufferers (El Khoury-Malhame et al., 2011a). Indeed, PTSD patients seem to have difficulties in avoiding the memorization of negative stimuli that would underlie an inhibition deficit for negative information (McNally et al., 1998).

Thirteen studies examined emotional memory by using recognition tests. Only four studies observed a greater memory in PTSD patients for negative stimuli, two of which highlighted a tendency for false recognition of negative stimuli. Tapia et al. (2012) used a Remember/Know paradigm during a recognition task. Participants were asked to recall stimuli, and if the participant was able to recall the stimuli in detail, a “remember” (conscious recollection) response was made. If recognition was not accompanied by such details, a “know” (familiarity) response was made. Results showed that PTSD patients had the same results as HC and A&D, but PTSD patients were more likely to “remember” negative words than to “know” them, compared to HC. According to Tapia et al. (2012), PTSD patients could pay greater attention to processing negative information. Guillery-Girard et al. (2013) used “negative perceptual” images (distressing images designed to evoke an emotional response), “negative conceptual” images (in which an emotional response arose from interpretation of the image) and “neutral” images. The authors divided the group of patients in two: PTSD patients with attentional deficit and PTSD patients without attentional deficit. In order to determine if PTSD patients had attentional deficit, the authors measured their performance using the neutral condition of the Oddball Paradigm test. The authors showed that PTSD adolescents with attentional deficit recognized fewer negative perceptual targets and produced more false negative conceptual recognitions than controls, while the PTSD adolescents without attentional impairment did not show any difference compared to HC. Attentional deficit in PTSD adolescents could have limited the effect of negative information on memory, and generated more false recognition. In other words, having an attentional deficit may simply mean that a strong emotional response is never generated from negative stimuli, because the subject doesn't pay as much attention as subjects without attentional impairment. This could lead to false recognitions of negative items which share similar conceptual properties. As in Guillery-Girard et al. (2013) and Thomaes et al. (2013) observed a trend for PTSD patients to make more false recognition of negative words than controls, but recognition of neutral words was similar between groups. Therefore, PTSD patients may be biased to recognize false negative stimuli. Other authors have also indicated that PTSD patients tend to recall more false negative information (Bremner et al., 2000; Brennen et al., 2007; Hayes et al., 2011). This may be due to a tendency for PTSD patients to remember overly general rather than detailed^***^ information (McNally, 1997). Another study assessed the “memory trade-off effect” (i.e., a greater memory for negative items superimposed onto neutral backgrounds) (Mickley Steinmetz et al., 2012). The authors reported that more of both positive and negative images were remembered than neutral for all participants. However, compared to TE, PTSD, and HC had a greater memory for emotional items (positive or negative) compared to neutral backgrounds, while there were no differences between neutral items in the foreground and neutral backgrounds. Nine other studies (Vrana et al., 1995; Chemtob et al., 1999; Moradi et al., 2000; Zoellner et al., 2003; Whalley et al., 2009; Brohawn et al., 2010; Baumann et al., 2013; Patel et al., 2016; Herzog et al., 2017) used recognition tests with different paradigms, and they did not find any differences between PTSD and control groups. Interestingly, three studies reported that PTSD patients had a greater memory for negative information in a free recall test, but there were no differences between PTSD and control groups in a recognition test (Vrana et al., 1995; Chemtob et al., 1999; Moradi et al., 2000). This is probably because recognition and free recall involve different processes: the former uses mainly episodic memory, and the latter uses associative memory (Gillund and Shiffrin, 1984). Two other studies also used free recall and recognition tasks, but emotional stimuli were equally recalled by PTSD and control groups (Zoellner et al., 2003; Herzog et al., 2017).

Three other studies used cued recall tests: either word-pair tasks, word-stem completion tasks, or both. These studies also produced contradictory results. A study found that PTSD and HC have the same emotional memory performance (Bremner et al., 2003) and two studies found a greater memory for negative information in PTSD groups (Zeitlin and McNally, 1991; Golier et al., 2003).

Golier et al. found that PTSD patients had poorer paired associate recall for trauma-related word pairs compared to neutral word pairs. Nevertheless, the authors observed a greater percentage of emotional word recollection in the PTSD group. PTSD patients recalled more words from the trauma-related word pairs than from the neutral pairs, while in control groups there were no differences. In contrast to Golier et al. Bremner et al. also used a word-pair task, but found no differences between PTSD and HC groups in enhanced memory for emotional vs. neutral word pairs. This can be explained by the fact that the authors used different word pair encoding conditions: Golier et al. used shallow and deep encoding whereas Bremner et al. did not make this distinction. Finally, in addition to a word pair task, Golier et al. used a word-stem completion task. After encoding neutral and trauma-related word-pairs, participants had to fill in the gaps between the first and last letters of words from both the previous word pair task and randomly chosen words. The results of this did not show any significant difference between the groups, but surprisingly, neutral word stems were correctly completed more often than trauma-related words in all groups. In Zeitlin and McNally, authors also used a word completion task. In contrast to Zeitlin and McNally (1991) and Golier et al. (2003) showed that PTSD patients recalled more combat-related words than other types of words compared to TE. These differences with TE were not found in other types of words. The authors also used a cued recall task and observed that PTSD patients have an equivalent memory for negative words, but memory impairment for other types of words. Several authors highlighted that memory impairment is present in PTSD (Tapia et al., 2007; Johnsen and Asbjørnsen, 2008). Despite this memory deficit, PTSD sufferers have an improved memory for negative words.

Taken together, these behavioral results tend to prove that PTSD is associated with a memory bias in favor of negative information, and less memorization of neutral information. Another study showed that the greater the severity of PTSD symptoms, the more the PTSD patient could recall fearful faces (Dickie et al., 2008). Thus, the severity of PTSD symptoms could play a role in memory bias toward negative stimuli: the greater the severity of PTSD symptoms, the more the PTSD patient could recall negative stimuli.

This specific memory processing in PTSD may be due to changes in memory when emotional stimuli are present, as well as attentional processes. Several authors discovered that PTSD patients display difficulties in disengaging their attention from negative stimuli (Bardeen and Orcutt, 2011; El Khoury-Malhame et al., 2011b; Aupperle et al., 2012). Therefore, negative stimuli capture their attention more, and are consequently better encoded and remembered.

This memory bias and difficulties in disengaging attention could also be interpreted as a reduction of the inhibition capacities specific to negative information.

Seven of the 18 studies did not find any behavioral differences between groups. Interestingly, however, most of these studies used neuroimaging which showed different areas of the brain being activated in PTSD patients compared to control groups, during emotional memory tasks. It is possible that the behavioral tasks used were more sensitive to neuroimaging than to behavioral assessment.

### Neuroimaging of Emotional Memory

In order to better understand emotional memory in PTSD sufferers, several researchers used neuroimaging during behavioral assessments. Among six studies: three studies researched different activations between PTSD and control groups during the encoding of emotional stimuli; two studies during retrieval of emotional stimuli; and one study explored both. Several studies reported that PTSD patients have different cerebral activities relative to controls when memorizing negative emotional words. These differences were observed mainly in the limbic areas and the frontal lobe.

Among those six reports, three studies observed differences in the cerebral activity of the hippocampus between PTSD patients and control participants: one study observed a decrease in hippocampal activity during retrieval, and two studies found an increase in hippocampal activity during encoding. Only Bremner et al. (2003) found a difference in hippocampal activity during retrieval. They used PET scans when PTSD and HC participants retrieved deeply encoded emotional and neutral word pairs. The authors did not find any differences in participants' memory capacity, but they observed a decreased blood flow in the left hippocampus. Compared to baseline, Thomaes et al. (2013) observed that while retrieving negative words that had just been encoded, the PTSD group showed a trend toward increased left hippocampus activity compared to HC. However, in the same situation, Brohawn et al. (2010) did not find significant differences in hippocampal activity in PTSD patients compared to control groups. Nevertheless, when the authors used another paradigm, the “Difference due to memory” paradigm (Dm), which is thought to reflect successful encoding processes, the PTSD group showed a greater activity in the negative Dm vs. the neutral Dm in the right hippocampus relative to the control group. Furthermore, they observed a positive correlation between amygdaloid and hippocampal activations during the encoding of negative vs. neutral pictures. This correlation was not found in the control group, suggesting an exaggerated functional connectivity between the hippocampus and amygdala in PTSD patients. A meta-analysis observed that functional connectivity between these two areas seems to be essential to emotional memory formation, specifically during encoding (Murty et al., 2010).

Other authors found differences in amygdaloid activity. Brohawn et al. (2010) found an increased activation in the left and right amygdala in the PTSD group during the encoding of negative compared to neutral pictures. This observation was not observed in the TE group, which may suggest that the amygdala was over-activated in the presence of negative stimuli. A review confirmed that amygdaloid activity increased in PTSD compared to HC participants (Shin and Liberzon, 2010). Furthermore, for PTSD patients, the amygdala could be implicated in attentional bias, with a notable orientation toward emotional stimuli (El Khoury-Malhame et al., 2011a). Also, Dickie et al. (2008) showed that the amygdala was positively correlated with the successful recall of fearful compared to neutral faces in PTSD patients. Another study selected in this review focused specifically on the amygdala during the encoding of negative vs. positive images: Patel et al. (2016), observed that during the successful encoding of negative images, the BLA is preferentially activated, compared to the CentroMedial Amygdala (CMA) in PTSD sufferers compared to TE participants. Mitra et al. (2005) demonstrated that stressful experiences can enhance synaptic connectivity in the BLA, which is involved in fear memory storage (Gale et al., 2004). Therefore, stressful events leading to PTSD may modify BLA neurons to respond to negative information. In contrast to Patel et al.'s hypothesis, a recent study did not find any difference in amygdaloid activation between groups (Herzog et al., 2017). These results may be explained by the level of attentional processing invested in the task. The level of attentional processing affected amygdaloid activity (Costafreda et al., 2008). Therefore, the cognitive demands could have been more intense in Herzog et al. (2017), compared to Patel et al. (2016), which could have led to greater inhibition in the amygdala.

Like previous authors, Herzog et al. (2017) showed an increased activation of limbic areas in PTSD participants. Specifically, they found a trend of increased activation in the right anterior insula and left dACC when PTSD patients were presented with trauma-related, compared to neutral and negative, words (Herzog et al., 2017). Thomaes et al. (2013) also observed a greater activation in the left dACC and the left ventral ACC (vACC) during the encoding of later-remembered negative words vs. baseline in their PTSD group. This greater activity in the right insula was also observed during the retrieval of deeply encoded emotional words, compared to deeply encoded neutral words in the PTSD group vs. the HC group (Bremner et al., 2003). The anterior insula and dACC are cerebral regions in the salience network. This network is activated in response to behaviorally salient events or stimuli (Ham et al., 2013), and its activation may explain a hypervigilance for trauma-related words in PTSD patients relative to the control group.

Authors of two selected studies also observed a greater activation in the PFC, notably the right dlPFC and left vmPFC, during the encoding of trauma-related words, although they did not find significant behavioral differences between PTSD and control participants (Thomaes et al., 2013; Herzog et al., 2017). The dlPFC is involved in cognitive and attentional control (Miller and Cohen, 2001; Blasi et al., 2007). Therefore, greater dlPFC activation may be linked to a greater attentional control in PTSD patients. Regarding the vmPFC, one review reported that the regulation of emotion, in particular the volitional control of negative emotion, is linked to an increase in vmPFC activity and a decrease in amygdala activity, which leads to a decrease in negative emotion (Koenigs and Grafman, 2009). The increase in vmPFC and dlPFC activation in both former studies (Thomaes et al., 2013; Herzog et al., 2017) may be caused by emotional regulation and cognitive control, which inhibit amygdala activation, in PTSD participants. This may explain why results showed no behavioral differences between groups in either study. Thomaes et al. (2013) made the assumption that the increase in activation in the vACC and dACC could be linked to difficulties in disengaging PTSD patients' attention from negative words. Thomaes et al. (2013) also investigated cerebral activity during a recognition task. They showed that patients had no differential activation compared to controls during the retrieval of negative words compared to baseline. However, the false recognition of negative words by PTSD patients was associated with enhanced activity in the left vlPFC and the lateral orbitofrontal cortex. The authors hypothesized that the increased activity in these areas was associated with impaired response inhibition due to memory intrusion.

Two studies indicated differences in cuneus or precuneus activation when retrieving emotional stimuli (Bremner et al., 2003; Whalley et al., 2009). Whalley et al. used an fMRI during a recognition task (involving neutral pictures that were superimposed onto emotional or neutral backgrounds). When negative hits were compared to neutral hits, they observed an increased activation in the right precuneus in the PTSD group relative to control groups. Bremner et al. using a word pair task, found a greater activation in the right cuneus in PTSD patients compared to HC groups. The cuneus and precuneus are known to be involved in visual processing and mental imagery recall (Fletcher et al., 1995; Cavanna and Trimble, 2006; Kroes et al., 2011). Thus, negative information in PTSD patients could involve stronger visual processing and mental imagery during retrieval, leading to a better retrieval of this information.

Taken together, the hyperactivation of the amygdala, cuneus/precuneus, and the hypoactivation or hyperactivation of hippocampal areas seems to be involved in the encoding and retrieving of negative information, and could also be linked to the symptoms of PTSD such as intrusion memories. Moreover, these cerebral areas could play a role in the attentional and memory processing of PTSD patients in favor of negative information, whether related to the traumatic event or not (Shin et al., 2006; El Khoury-Malhame et al., 2011b; Hayes et al., 2011).

The selected neuroimaging studies also showed a hyperactivation of the PFC in PTSD patients during a task involving negative stimuli. These alterations could underlie hypervigilance for negative information.

### Neurobiology and Emotional Memory

Only one study assessed the role of corticosteroids and noradrenaline in PTSD during an emotional memory task (Nicholson et al., 2014). Although neither noradrenaline nor cortisol were predictors of negative stimuli recall, after viewing negative images, PTSD patients had increased noradrenaline saliva levels relative to the HC group. Nevertheless, contradictory to Nicholson et al. (2014), previous studies had observed that stressful or negative stimuli generate noradrenaline release into the amygdala, which enhances the memorization of emotional information (O'Carroll et al., 1999; Roozendaal et al., 2009).

## Limitations

The two main limitations of this review are its small sample size, as well as the different types of tasks, paradigms and stimuli used. Furthermore, seven studies did not include a TE control group, which rules out the possibility of determining whether memory performance is related to trauma exposure or PTSD.

Another limitation is the discrepancy between control groups, which can result in bias when comparing studies. For instance, seven publications were restricted to female PTSD patients, and three studies were restricted to males. Some authors suggest gender-related differences in emotional memory (Canli et al., 2002), and one review reported different prefrontal and limbic activations between men and women during an emotional memory task (Hamann and Canli, 2004). Furthermore, the mean and range of the delay between exposure to the traumatic event and the start of the study were not specified in several of the selected studies. Further limitations were a possible risk of bias due to the absence of blinding for assessors in all of the selected reports, as well as differences in control group matching, notably in terms of cognitive ability or current medication.

## Conclusion

The overall goal of this review is to explore cognitive differences in processing emotional stimuli compared to neutral stimuli in PTSD sufferers. Eighteen controlled studies were selected, including a total of 387 patients with current PTSD.

Among the 18 studies selected, 11 studies observed differences in emotional memory performance in PTSD patients. PTSD patients seem to have more memorization of negative information, and less memorization of neutral information compared to control groups. Some studies also found a trend to false recognition of negative information, a greater ability to “remember” negative information, or difficulties to avoid memorization of negative information. The implication of a memory bias in favor of negative information also seems to be linked to an attentional bias for negative information, as well as a deficit in inhibition function, which would help patients to avoid memorization of negative information.

Most of the studies that used neuroimaging did not find significant behavioral results. This could be due to the behavioral tasks being more sensitive to neuroimaging than to behavioral assessment. During emotional memory tasks, different cerebral areas were activated in PTSD patients compared to control groups. The neuroimaging in these studies showed a hyperactivation of the amygdala and PFC, as well as abnormal activity of hippocampal areas. These three areas are involved in encoding and retrieving negative information, and are also be linked to the symptoms of PTSD, such as intrusion memories and hypervigilance.

Over the last few years, public health institutions have tended to mitigate the problem of PTSD by recommending the use of therapies such as Eye Movement Desensitization, Reprocessing Therapy, or Cognitive Behavioral Therapy (American Psychiatric Association, 2004; National Institute for Health Clinical Excellence, 2005; World Health Organization, 2013). Nevertheless, current screening and treatment could be more effective with more information about cognitive and emotional processing in PTSD. This review, which deals with the interaction between emotional and memory processes in PTSD symptoms, could help clinicians improve PTSD therapy programs and patient monitoring, and help to create new therapeutic tools (i.e., psychoeducational programs). This review could also provide researchers with methodological information on emotional memory studies. For instance, matching control groups to PTSD groups for cognitive ability, gender, and medication seems to be a necessity, in order to avoid bias.

Future studies could evaluate the impact of emotion on memory in PTSD patients, after their symptoms have improved. Effective treatment in PTSD, such as EMDR therapy, could modify and normalize the impact of emotion in memory. Consequently, emotional memory could be linked to the improvement of clinical symptomatology in PTSD.

## Author Contributions

FD searched using the terms to identify relevant emotional memory studies in PTSD population. Titles and abstracts were screened by FD in order to remove duplicates. Then FD and CI assessed independently titles and abstracts and articles. CI and FD excluded articles if both reviewers decided that the articles clearly did not meet criteria. In case of disagreement, the two reviewers had to reach a consensus. CI and DJ read and made corrections the entire manuscript.

### Conflict of Interest Statement

The authors declare that the research was conducted in the absence of any commercial or financial relationships that could be construed as a potential conflict of interest.

## Appendix

Search strategy: ScienceDirect Post-Traumatic Stress Disorder AND Emotional memory (Search results: 43)

Post-Traumatic Stress Disorder AND Emotion AND memory (Search results: 56)

Post-Traumatic Stress Disorder AND Emotion AND episodic memory (Search results: 5)

Post-Traumatic Stress Disorder AND Emotional” AND episodic memory (Search results: 7)

Posttraumatic Stress Disorder AND Emotional memory (Search results:43)

Posttraumatic Stress Disorder AND Emotion AND memory (Search results: 94)

Posttraumatic Stress Disorder AND Emotion AND episodic memory (Search results: 4)

Posttraumatic Stress Disorder AND Emotional AND episodic memory (Search results:7)
